# Homeopathic Preparations of Quartz, Sulfur and Copper Sulfate Assessed by UV-Spectroscopy

**DOI:** 10.1093/ecam/nep036

**Published:** 2011-04-14

**Authors:** Ursula Wolf, Martin Wolf, Peter Heusser, André Thurneysen, Stephan Baumgartner

**Affiliations:** ^1^Institute of Complementary Medicine KIKOM, University of Bern, 3010 Bern, Switzerland; ^2^National High Magnetic Field Laboratory (NHMFL), Florida State University, Tallahassee, FL 32310, USA; ^3^Institute Hiscia, 4144 Arlesheim, Switzerland

## Abstract

Homeopathic preparations are used in homeopathy and anthroposophic medicine. Although there is evidence of effectiveness in several clinical studies, including double-blinded randomized controlled trials, their nature and mode of action could not be explained with current scientific approaches yet. Several physical methods have already been applied to investigate homeopathic preparations but it is yet unclear which methods are best suited to identify characteristic physicochemical properties of homeopathic preparations. The aim of this study was to investigate homeopathic preparations with UV-spectroscopy. In a blinded, randomized, controlled experiment homeopathic preparations of copper sulfate (CuSO_4_; 11c–30c), quartz (SiO_2_; 10c–30c, i.e., centesimal dilution steps) and sulfur (S; 11×–30×, i.e., decimal dilution steps) and controls (one-time succussed diluent) were investigated using UV-spectroscopy and tested for contamination by inductively coupled plasma mass spectrometry (ICP-MS). The UV transmission for homeopathic preparations of CuSO_4_ preparations was significantly lower than in controls. The transmission seemed to be also lower for both SiO_2_ and S, but not significant. The mean effect size (95% confidence interval) was similar for the homeopathic preparations: CuSO_4_ (pooled data) 0.0544% (0.0260–0.0827%), SiO_2_ 0.0323% (–0.0064% to 0.0710%) and S 0.0281% (–0.0520% to 0.1082%). UV transmission values of homeopathic preparations had a significantly higher variability compared to controls. In none of the samples the concentration of any element analyzed by ICP-MS exceeded 100 ppb. Lower transmission of UV light may indicate that homeopathic preparations are less structured or more dynamic than their succussed pure solvent.

## 1. Introduction

Homeopathy and anthroposophic medicine are complementary medical systems that use high or ultra-high dilutions, also known as homeopathic preparations or homeopathic potencies. These homeopathic preparations are prepared by logarithmically diluting and succussing a mother tincture, typically in water or water-ethanol mixtures. The dilution level that will ultimately be used may be beyond the Avogadro number, for example, the probability for even a single molecule of the mother tincture to be present in the dilution is virtually zero. Although several randomized placebo-controlled double-blind clinical trials reported effects of homeopathic preparations superior to placebo [1–8], their clinical effectiveness was disputed by a recent meta-analysis [6] that launched a debate and earned public attention. Subsequently, several authors, including statisticians, detected fundamental methodological problems with this meta-analysis [9–11]. A recent health technology assessment [8] reports that clinical effectiveness of homeopathy is supported by evidence. Thus, the effectiveness of homeopathy is still a subject of debate.

It is often argued that the effects of homeopathic dilutions are either unspecific or placebo, since common scientific theories and models cannot account for any specific effects of homeopathic dilutions.

Within the last years, several working hypotheses have been developed to reveal the mode of action of homeopathic preparations but none of them has been validated so far [12–21]. Therefore, knowledge of the nature of homeopathic preparations is yet insufficient.

In addition, considering the conditions of modern life, the question about the stability of homeopathic preparations arises. It is yet unclear whether certain factors such as pharmaceutical procedures (e.g., autoclavation), artificial magnetism, ionizing radiation (e.g., scanner at airports, train stations) or devices emitting non-ionizing radiation (e.g., mobile communication) might affect the stability and quality of homeopathic preparations. Given these uncertainties, there clearly is need for further research.

One important step is the investigation of physical properties of homeopathic preparations using standard techniques. Previous studies (for a review see [22]) of physical properties of homeopathic preparations included measurements of electrical conductivity, electrical resistance, dielectric constant, thermodynamic properties [23], thermoluminescence [24] and methods such as nuclear magnetic resonance (NMR), spectroscopy and relaxation [25–30], Raman-spectroscopy and ultraviolet (UV)-spectroscopy [31–36]. In several previous studies, differences in UV absorption of homeopathic preparations and controls were observed. Lower transmission values for homeopathic preparations of Atropa Belladonna [32] and Nux vomica [34] were found, while another study did not show obvious differences [35]. Relatively large differences between succussed and unsuccussed media were observed [33, 35]. More experimental evidence is needed.

In addition, it is not yet clear which measurement methods are best suited to determine specific physicochemical properties of homeopathic preparations (in case there are any). UV-spectroscopy is a little investigated method that yielded promising results in own previous pilot measurements.

The aim of our study was to investigate homeopathic preparations of copper sulfate, sulfur and quartz with UV-spectroscopy and compare them to controls.

## 2. Methods

### 2.1. Laboratories and Clean Room

The experiments were carried out at two laboratories in the USA, at the University of Illinois at Urbana Champaign (Lab 1) and at the National High Magnetic Field Laboratory, Tallahassee, FL (Lab 2).

Lab 1 was a standard wet laboratory; while in Lab 2 all samples were prepared in a metal-free class 100 HEPA-(High Efficiency Particulate Air) filtered clean room. Clean flow boxes had class 5. It was intended to test whether the two different laboratories and their conditions may have an influence on the results.

### 2.2. Water Preparation

In Lab 1 we used distilled water as potentization medium. In Lab 2 water was prepared according to standard procedures in trace analytics. De-ionized water (DI-water) was prepared from tap water using two ion-exchange columns (Culligan, Northbrook, IL, USA) for a first de-ionization and a subsequent Millipore system (Super-Q water purification system with four cartridges: 1. Super-C for organic removal, 2. Ion-Ex, and 3. Ion-Ex for inorganic removal, and 4. Durapore for bacteria and particle removal), resulting in water of 18 MΩcm. Quartz distilled water (QD-water) was prepared by subsequent sub-boiling distillation of the DI-water (Seastar Chemicals Inc., Sidney BC, Canada).

### 2.3. Chemicals

In Lab 1 we used copper sulfate (CuSO_4_ *·*5H_2_O) from Weleda AG, Arlesheim, Switzerland.

In Lab 2 hydrochloric acid (HCl) was sub-boiling double-distilled HCl, prepared from reagent grade HCl (certified ACS PLUS, normality 12.1, A 2005–212, from Fisher Scientific, Fairlawn NJ, USA). Nitric acid (HNO_3_) was twice two-bottle distilled HNO_3_, prepared from reagent grade HNO_3_ (certified ACS PLUS, normality 15.8, A 1445–212, from Fisher Scientific, Fairlawn NJ, USA). Ethanol used was Ethyl Alcohol USP, Absolute-200 Proof (Aaper Alcohol and Chemical Co., Shelbyville, USA). Lactose was ordered from Dixa AG, St. Gallen, Switzerland, quartz powder (SiO_2_) from Weleda AG, Schwaebisch Gmuend, Germany, copper sulfate (CuSO_4_ *·*5H_2_O) from Weleda AG, Arlesheim, Switzerland and sublimed sulfur (S_8_ in the following abbreviated as S) from Phytomed AG, Hasle/Rueegsau, Switzerland. ICP-MS standards were obtained from High-Purity-Standards, Charleston SC, USA.

### 2.4. Vessels

In Lab 1 the potentization vessels were 100-ml narrow-necked bottles with conical shoulder, made from boro-silicate glass, hydrolytic class 1 and thus highly resistant to ion leaching (DURAN, Schott, from VWR International, Dietikon, Switzerland). In Lab 1 the vessels were cleaned using detergent, alcohol and distilled water. In a control measurement the vessels were filled with water that was succussed and transmission was measured using UV-spectroscopy. The data were analyzed and no relevant outliers were detected.

In Lab 2 vessels for all liquids were 500-ml narrow-necked bottles with conical shoulder, also made from boro-silicate glass, hydrolytic class 1 (DURAN, Schott, from VWR International, Dietikon, Switzerland). All 40 vessels used were numbered permanently in order to be able to retrace the use of every individual vessel during the entire study. In a control measurement the vessels were filled with water, then the water was succussed in these vessels and transmission of UV light was measured. The data were analyzed, and no relevant outliers were detected. After production of one series of homeopathic preparations and the corresponding controls, all vessels were cleaned (see below) and re-used in randomized allocation for the next series. Trituration (potentization of solid compounds) was performed with a porcelain mortar and pestle.

For the ICP-MS measurement 4-ml polypropylene vials (Omni vials Polypropylene (PP), Cole-Parmer, Vernon Hills IL, USA) were used.

To minimize ion release from the vessel walls, all vessels were pretreated in Lab 2 as customary in inorganic trace analytics. In Lab 2 the treatment of the potentization vessels before the first use included: Rinse 3× with DI-water, fill 1/4 of height with 1.2 N HCl (12.1 N, Fisher Scientific, 1 : 10 diluted with QD-water), put vessels on hot plate (125°C) for 8 h in a clean flow box, remove HCl, rinse 3× with DI-water, rinse 3× with QD-water.

In Lab 2 cleaning of the potentization vessels before S potentization consisted of: rinsing 3× with QD-water. Potentization vessels before CuSO_4_ potentization: rinsing 3× with DI-water and 3× with QD-water. Vessels having contained homeopathic dilutions with concentrations higher than 10–10 were rinsed 6× with DI-water and 3× with QD-water.

ICP-MS-vials and pipette tips: 24 h in 7.9 N HNO_3_ (15.8 N, Fisher Scientific, 1 : 1 diluted with QD-water) on hot plate (100°C), rinse 3× DI-water, rinse 3× QD-water.

### 2.5. Production of Homeopathic Preparations and Controls

In order to complement earlier investigations using nuclear magnetic resonance, we decided to investigate homeopathic preparations of quartz (SiO_2_) like Demangeat [25] and sulfur (S_8_) like Weingärtner [30]. Copper sulfate (CuSO_4_) was tested, because it emerged as promising candidate in own pilot experiments (unpublished data).

Homeopathic preparations were produced in such a way that they met current legal regulation for homeopathic remedies [37] and controls. As controls we used succussed potentization medium. This control accounts for all unspecific physicochemical effects such as increased ion and air dissolution, air suspension, and radical formation, compared to unsuccussed solvent [38]. We did not use potentized solvent in this study because specific effects have been reported in biological models [39–41].

Quartz (SiO_2_) and copper sulfate (CuSO_4_) were prepared as c-preparations (centesimal homeopathic preparations, 100-fold dilution), S as x-preparations (decimal homeopathic preparations, 10-fold dilution) in order to allow a comparison with previous investigations [25, 26, 30]. In Lab 1 only CuSO_4_ and in Lab 2 all three types of homeopathic preparations were produced.

In Lab 1 the potentization medium was distilled water, while in Lab 2 it was quartz distilled water with 1% ethanol.

Trituration (potentization of solid compounds) was performed by hand for 60 min according to standard pharmaceutical procedures (prescription no. 6 of the German Homeopathic Pharmacopoeia [37]). One gram SiO_2_ powder was triturated with 99 g lactose with mortar and pestle to obtain SiO_2_ 1c. SiO_2_ 2c and 3c were prepared analogously from SiO_2_ 1c or 2c, respectively. Ten grams S powder were triturated with 90 g lactose to obtain S 1×. S 2×, 3× up to 6× were prepared analogously from S 1×, 2×, up to 5×, respectively.

Potentization was performed by hand according to standard pharmaceutical procedures with the multiple glass method [37]. Potentization was done by horizontally shaking the vessel at a rate of about 2.7 Hz for 4 min before each dilution. For CuSO_4_, the first homeopathic preparation level (1c) was made by dissolving 0.2 g (Lab 1) or 2 g (Lab 2) of CuSO_4_ in 20 ml (Lab 1) or 200 ml (Lab 2) potentization medium at 37°C. For the next potentization step, 1% of fluid was pipetted into another potentization bottle and succussed as described earlier. All further potentization levels were prepared analogously. For SiO_2_, liquid potentization started with the dissolution of 2 g SiO_2_ trituration 3c in 200 ml QD-water with 1% ethanol. Shaking resulted in SiO_2_ 4c. All further liquid potentization levels were prepared as described earlier for CuSO_4_. For S, liquid potentization started with the dissolution of 2 g S trituration 6× in 200 ml QD-water with 1% ethanol. Shaking resulted in S 7×. All further liquid potentization levels were prepared analogously as described earlier, but with a dilution ratio of 1 : 9 (instead of 1 : 99). All homeopathic preparations and controls of a given set (SiO_2_, S or CuSO_4_) were prepared from the same batch of QD-water with 1% ethanol.

For each set of homeopathic preparations (SiO_2_, S or CuSO_4_), 4 (Lab 1) and 10, respectively (Lab 2), independent controls were produced, using the same potentization medium and shaken equally to the homeopathic preparations. This procedure resulted in a preparation called “agitated potentization medium”. In Lab 2, five of the controls were prepared before the preparation of the homeopathic preparations and five controls after, in order to control for a possible cross-contamination and other interference in the course of the production process.

Randomization was effectuated through randomly (random numbers from a computer) allocating the numbered potentization vessels to the homeopathic preparation levels or controls to be produced. After preparation, the bottles were placed in random order, and the codes were kept secret on a hidden allocation list on paper. Thus, the experiment was blinded. Codes were only revealed after the end of the measurements and data reduction. The measured homeopathic preparations are diluted to such a degree that they cannot be distinguished from controls by any of the human senses.

### 2.6. ICP-MS Measurements

Samples of 3 ml of each homeopathic preparation and control were pipetted into ICP-MS-vials to which 15 *μ*l internal standard (^45^Sc, ^74^Ge, ^115^In, ^205^Tl, 1 ppb each) and 30 *μ*l 15.8 N HNO_3_ were added. Samples were prepared in the clean room and sealed with a cap. Samples were transferred to the ICP-MS-autosampler and opened only under the protection hood of the sampler.

For analysis, a Sector ICP-MS Finnigan MAT Element (Thermo Electron, Karlsruhe, Germany) with PFA inlet system, Teflon spray chamber, and PFA nebulizer with a flow rate of 100 *μ*l/min was used. The system was run with guard electrode in operational mode. Analyzed elements were ^7^Li, ^11^B, ^23^Na, ^24^Mg, ^27^Al, ^28^Si, ^44^Ca, ^48^Ti, ^56^Fe, ^65^Cu, ^66^Zn, ^85^Rb, ^88^Sr, ^133^Cs, ^137^Ba and ^208^Pb, measured either in low- or medium-resolution mode. They represent the most common impurities known to trace analytics.

Samples were measured in random order in runs of 10 samples. Blank and external standard samples (all analyzed elements in a concentration of 1 ppb) were measured at the beginning, in the middle and at the end of each run.

After measurement, data reduction was performed according to standard procedures of analytical chemistry [42, 43]. For each run, the corresponding calibration curve was based upon the values of the external standard and of the blank (*n* = 3 each). The inverted calibration curve was used to calculate effective concentrations and the error (95% confidence limits) for all samples. Detection limit determination was based upon the standard deviation of the blank for alpha = beta = 5%.

### 2.7. UV-Spectroscopy Measurements

At Lab 1, a Perkin-Elmer *λ*14 and, at Lab 2, a Perkin-Elmer *λ*3B UV-spectrometer and, in both laboratories, high-quality quartz cuvettes (Hellma quartz Suprasil 1 cm) were used. These double-beam UV spectrometers are comparable. The light transmission of all samples was measured from 190 to 290 nm. Each measurement was repeated three (Lab 1) or five (Lab 2) times, respectively. In Lab 1, the reference was air and, in Lab 2, a cuvette filled with distilled water. Both UV spectrometers were turned on 1 h before the measurement to allow a warm-up of the instruments. In pilot studies, wavelength calibration and handling were tested to optimize reproducibility. Both instruments scanned with a speed set at 120 nm/min. Every 35th (Lab 1) or 10th (Lab 2) measurement, respectively, was without any sample inserted in the UV spectrometer, followed by one with a sample of the cleaning water. After measuring a sample, the cuvette was cleaned twice with distilled water (Lab 1) or quartz distilled water (Lab 2) and shaken out before filling it with the next sample. When filling the cuvettes, care was given to avoid bubbles and cuvettes were visually inspected for bubbles. The cuvettes were filled using pipettes with a standardized volume.

Table 1 illustrates the production and measurement process and displays, where the homeopathic dilutions and controls were prepared and measured and at what age and with which instrument.

### 2.8. Data Analysis

Data was averaged across the three and five repetitions, respectively, and across two bands, that is, from 190 to 290 nm and from 215 to 290 nm. In the band, from 190 to 215 nm, measurements are less stable since these wavelengths are at the border of the measuring range of the UV spectrometer and, consequently, between 190 and 215 nm, the instrumental noise is higher than above 215 nm. But since the effects may possibly be stronger below 215 nm, because the UV absorption of water is higher here, the band below 215 nm was once included and once left out.

All statistics were calculated in SPSS 15.0. The difference in light transmission between homeopathic preparations and controls was tested using a *t*-test, which does not assume equal variances. Equality of variances was analyzed by the Levene's test. To pool data across the different series, it was necessary to adjust the mean transmission between the series, which depends on the baseline setting of the instrument and reference. Since a potential effect is always given as a proportion of the transmission, each value was scaled in the following way:

(1) $$\text{Scaled}\_\text{value}=\frac{100\times \left(\text{Original}\_\text{value}-\text{Mean}\_\text{of}\_\text{value}\right)}{\text{Mean}\_\text{of}\_\text{value}}.$$

Mean_of_controls refers to the mean of the controls of that specific series of measurements. Statistics of pooled values were calculated for all five series of measurements, for the three CuSO_4_ series and for the measurements in Lab 2.

ANOVA was used to analyze variability between and within samples for CuSO_4_.

In addition, between the different CuSO_4_ series, the correlation (Pearson & Spearman) was determined.

## 3. Results

**Figure 1:**
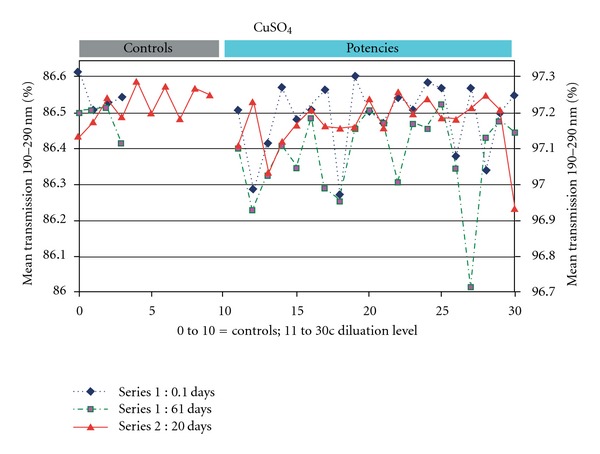
Copper sulfate (CuSO_4_) homeopathic preparations and controls: The averaged UV transmissions from 190 to 290 nm of the two separate preparations (series 1 and 2) are displayed in percentage. The controls are on the left, the homeopathic preparations on the right side. For the Series 1 with age 61 days and Series 2 the difference between homeopathic preparations and controls was significant.

**Figure 2:**
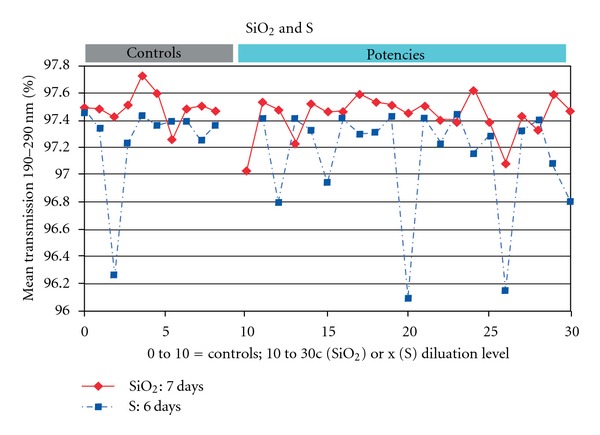
The UV transmissions of the quartz (SiO_2_) and sulfur (S) homeopathic preparations and their respective controls.

In Figures 1 and 2 and Table 2 the results of the measurements and statistics are displayed.

The UV transmission for CuSO_4_ preparations was significantly lower than in controls in two out of three measurements for the band of 190–290 nm, and in one out of three measurements (Lab 2) for the band of 215–290 nm. Pooling all three CuSO_4_ measurements led to highly significant (*P* < .001) differences between homeopathic preparations and controls for both bands.

The transmission was also lower for both SiO_2_ and S, but not significant. Pooling all measurements (CuSO_4_, SiO_2_ and S) again led to significant differences between homeopathic preparations and controls for both bands. Pooling all the measurements of Lab 2 yielded significant differences for the band of 215–290 nm.

The effect size was remarkably similar for the homeopathic preparations of all substances, that is, the difference between homeopathic preparations and controls ranged from 0.0457–0.1257% for the band of 190–290 nm and from 0.0281–0.0656%.

UV transmission values between homeopathic preparations in terms of the SD had a higher variability in homeopathic preparations compared to controls. These differences in SD were not significant for any series by itself. However, when the CuSO_4_ data were pooled, the mean SD was a factor 2.53 (*P* = .017) larger for the homeopathic preparations, compared to the controls for data from 190 to 290 nm. The result was similar for 215–290 nm (factor 2.27; *P* = .025).

ANOVA for the three CuSO_4_ series showed for data between 190–290 nm a between-group (homeopathic potency level and control) mean square of 0.0192 (*P* = .056), which is larger than the within-group square of 0.0116. For 215–290 nm the between-groups mean square of 0.0103 was significant (*P* = .040) and also larger than the within-group square of 0.0059.

There were no significant correlations between the three CuSO_4_ series, which indicates that there is no specific pattern of higher or lower transmission for different potency levels.

The ICP-MS analysis showed that the samples produced were highly pure, that is, in Lab 1, the concentrations of all ions were <100 ppb and, in Lab 2, <10 ppb. In the following, the elements are sorted according to their mean concentrations in decreasing order.

In Lab 1, the mean concentrations of elements between 21 and 10 ppb were: ^11^B, ^24^Mg, ^23^Na, ^44^Ca; between 10 and 1 ppb were: ^28^Si, ^65^Cu, ^27^Al; and below 1 ppb were: ^66^Zn, ^208^Pb, ^137^Ba, ^88^Sr, ^48^Ti, ^56^Fe, ^85^Rb, ^7^Li and ^133^Cs.

In Lab 2, most elements were below the detection limit of approximately 1 ppb. Only ^11^B and ^23^Na were detectable at mean concentrations between 1 and 4 ppb. The CuSO_4_ samples were particularly clean, that is, only ^11^B was detectable at concentrations <2 ppb.

Thus, the use of the clean room and sophisticated procedures improved the purity by one order of magnitude. There were no significant differences in the ion concentrations between homeopathic preparations and controls.

## 4. Discussion

In our results we found significant differences, that is, a lower transmission of UV light, in two series of homeopathic CuSO_4_ preparations. Also for potentized SiO_2_ and S we observed differences in transmission pointing in the same direction although they were not significant. The question arises whether these differences could be due to artifacts, that is, trivial physicochemical explanations? To clarify this, the following aspects need to be considered.

### 4.1. Instrument

Both UV spectrometers were double-beam instruments, which enhance the stability of the measurements and thus increase the reproducibility of the measurements.

The instrumental drift that was monitored throughout the measurements was negligible and it was not necessary to corrected for it in the data analysis. The drift during the CuSO_4_ measurements was even smaller than for the other homeopathic preparations. Therefore, the results are not biased by an instrumental drift.

The humidity of the air may influence UV transmission measurements because water is absorbing light. Since the humidity affects the measurement and the reference beam of double-beam spectrometers in the same way, its influence is negligible. In addition, the measurements were carried out under stable weather conditions and did not exceed 4 h; a time in which a considerable change in humidity in an air conditioned laboratory is minimal.

Room temperature may influence UV-spectroscopy measurements. However, the room temperature was constant throughout the measurements.

The amount of dissolved oxygen in water, that is, the diluent, affects its UV-spectroscopic absorption properties, but since this factor affects both controls and homeopathic preparations in the same way, it can be ruled out.

The reproducibility of the measurements was good; in a pilot study in Lab 2, the error for the range between 190 and 290 nm was 0.0059% (empty cuvette) and 0.119% (cuvette filled with water and refilled for each measurement) and for the range between 215 and 290 nm was 0.0045% (empty cuvette) and 0.076% (cuvette filled with water and refilled for each measurement). This shows that the reproducibility of the instrument is higher and that refilling the cuvette affects the reproducibility more than the instrumental factors. This high reproducibility was also demonstrated by the fact that measurements carried out in two different laboratories with two different instruments and two different homeopathic preparations of CuSO_4_ led to similar effects.

Most importantly, all mentioned factors would have affected both homeopathic preparations and controls in the same way and could be, even if they had occurred, ruled out due to the randomization.

### 4.2. Contamination

To test whether the samples were contaminated by traces of dust or inorganic contaminants, all samples were also measured by ICP-MS. These measurements showed that the contamination was negligible for both Lab 1 and Lab 2 (<100 ppb, resp., <10 ppb for all ions, data not shown). In addition, contamination in Lab 2 was particularly low for CuSO_4_ homeopathic preparations (<2.6 ppb for all ions). Previously, quantitative concentrations of contaminating ions such as Na, Si, Mg, Al, Li and Fe were reported for brown glass bottles [44]. In Lab 1 the concentrations of Na was 33 times, Si 208 times, Al 2.3 times, Li 538 times and Fe 211 times lower in our study, compared to brown glass. Only Mg was slightly higher in our study by a factor 1.5. In Lab 2, Na, the only detectable contaminant, which can be compared to these data, had a 155-times lower concentration compared to the brown glass bottles. Thus, the preparations in our study were highly pure, in particular for Lab 2. Although the preparations in Lab 2 were much purer than in Lab 1, the difference between homeopathic preparations and controls in UV transmission values was quite similar and statistically significant in both cases.

Therefore, the detected differences in transmission are not due to contamination. Since this factor applies to both controls and homeopathic preparations in the same way, it can be excluded.

### 4.3. Experimenters Influence

All samples were blinded and blinding was only disclosed after data analysis was completed. Moreover, all measurements were carried out in a randomized order. Therefore we can exclude bias caused by the experimenter.

### 4.4. Leaching

In Lab 1 we used vessels of hydrolytic class 1, which are highly resistant to leaching. Although they were not pre-treated as in Lab 2 the homeopathic preparations and controls were very pure with a concentration of ions <100 ppb.

Since all glassware at Lab 2 that was used for the preparation and storage of the samples was treated according to trace analytics prior to their use, leaching of ions from the vessel walls is minute. The only vessels that were not treated according to the trace analytics protocol were the cuvettes. The residence time of a sample in a cuvette, however, was less than 2 min. We consider this time to be too short to induce a considerable leaching. Had a leaching been taken place by the repeated filling and emptying of the cuvettes during the measurements we would have noticed that as a drift in the values. Moreover, since the measurements were done in a randomized order, and a possible leaching effect would affect both homeopathic preparations and controls in the same way. Therefore, this cannot explain the differences found.

Since there are no trivial artifacts that could lead to the significant differences between homeopathic preparations and controls what does this effect mean? The UV transmission values were lower and significantly different, compared to controls for CuSO_4_ homeopathic preparations that were measured more than two weeks after preparation. For SiO_2_, S and the first CuSO_4_ series, which were all measured within two weeks after the preparation, all had lower transmission values for homeopathic preparations compared to controls, but these differences were not significant.

### 4.5. Dynamization Hypothesis

It seems that homeopathic preparations have a lower UV transmission compared to controls. A lower transmission in UV-spectroscopy corresponds to a higher absorption of light. In general, absorption is explained either as an electron being moved to a higher energy level by a quantum of light or by an increase in the vibrational energy of a molecule. The sharp absorption edge between 160 and 200 nm corresponds to an electronic transition between non-bonding and anti-bonding states (*n* → σ*) of electrons located in the lone pairs on the oxygen atom in the water molecule [45]. The non-bonding electrons involved in this transition are the same electrons that act as hydrogen acceptors during formation of inter-molecular hydrogen bonds. Thus the absorption also depends on the structure of water: higher temperatures (implying weaker H-bonds) lead to increased UV absorption [46]. The lower transmission values indicate that the diluent is less structured or more dynamic after homeopathic potentization. Such a phenomenon could be caused by a non-thermal metastable energy state. The possibility of such metastable states in a liquid in the context of current water structure theories remains to be explored.

### 4.6. Hypotheses of Specific Homeopathic Drug Effects

Several working hypotheses to describe the mode of action of homeopathic preparations have been proposed including theories based on placebo, water structure (clusters or clathrates), silica contamination and entanglement models of quantum theory, but none of these has been validated so far [12–21, 47–49].

Placebo can be ruled out in our study as well as silica contamination.

Some studies and theories suggest that there might be particular structures (clusters) in water, which are causative for the homeopathic effect [49, 50]. However, recent investigations with high-field ^1^H-NMR-spectroscopy did not yield any evidence for stable water clusters (life span > ms) within liquid homeopathic remedies [27, 28]. On the contrary, high-quality studies using NMR relaxation [25, 26] as well as our results seem to indicate the opposite—less structured water.

The entanglement theory, which is based on a weak quantum theory, is one possibility explored by many authors [12–16, 18, 20, 21]. Again, this theory has not been proven and, in particular, it is unknown how information should be transferred by entanglement effects. The models are not developed far enough to predict how entanglement would affect UV transmission.

We would like to emphasize that at the moment it is unclear, whether any theoretical model is able to explain our findings. Therefore, the mode of action of homeopathic preparations remains unclear. The observation in our experiment that the homeopathic preparations are less structured may serve as an indicator for future models on homeopathic dilutions.

### 4.7. Other Investigations of Homeopathic Preparations with UV-Spectroscopy

In several previous studies differences in UV absorption of homeopathic preparations and controls were observed. Effects pointing at the same direction were reported in one study [32], where for homeopathic preparations of Atropa Belladonna 30× and 200×, a higher absorption between 190 and 220 nm compared to controls (probably unsuccussed solvent) was measured and was interpreted as a dynamization of the homeopathic preparations. In another study [35], Lycopodium clavatum 6c, 12c and 100c, were compared to solvent with (3c and 6c) and without succussion. No statistics were presented. From the figures no differences between homeopathic preparations and succussed controls were visible. Unsuccussed controls clearly looked different. In addition, the experiment was repeated and there were differences between the two sets, which were presumed to be due to contaminants from two different batches of solvent used for the two experiments [35]. However, since the measurements of the unsuccussed solvent were similar between the two sets, it is more likely that the difference between the two sets is related to the succussion process. This again emphasizes the need to prepare succussed controls and corresponds to our experience (unpublished data). Homeopathic preparations of Nux vomica 30c succussed, Nux vomica 30c only diluted but not succussed and the solvent alone were measured [34] and a considerably higher absorbance for both Nux vomica preparations compared to the solvent and a similar absorbance with a slight difference between the Nux vomica preparations reported. No statistical analysis (variability or significance) was provided, probably because measurements were not repeated. Another study [33] confirmed the relatively large differences between succussed and unsuccussed medium. Also clear differences between two different homeopathic preparations (NaCl and Nux vomica) were found, but no statistical analysis was provided to support this finding although the measurements were repeated 10 times. Thus, in general previous studies report higher absorption, which corresponds to lower UV light transmission for homeopathic preparations, which is in agreement with our findings.

### 4.8. Variability

The transmission values for the homeopathic preparations had a higher variability between homeopathic preparations in comparison with the controls. Although this effect was not significant for any of the homeopathic preparation series by itself, it was significant once the CuSO_4_ data was pooled. The higher variability could indicate that the degree of dynamization depends on the homeopathic preparation level. This may indicate that a homeopathic preparation series expresses peaks and troughs, an effect that was discovered earlier [51]. In the same report, the peak and troughs depending on the dilution levels were found to shift slightly from one preparation to the next, which may explain, why no significant correlation was found between our preparations.

### 4.9. Trituration and Time Course

SiO_2_ and S homeopathic preparations were prepared from triturations, while CuSO_4_ directly from the dissolved mother substance. Since significant differences were only observed in CuSO_4_ preparations (Tbale 2), the question may arise whether the trituration process has an influence on the UV transmission of correspondingly prepared homeopathic samples. However, the effect sizes (differences between potencies and controls) for UV transmissions were quite similar for SiO_2_ and S compared to CuSO_4_ (Table 2). Thus there is no obvious support for an effect of the trituration in the present study. However, it would be valuable to address this question by explicitly designed investigations, for example, by comparison of homeopathic samples prepared from triturated or directly dissolved copper sulfate.

The measurements of CuSO_4_ immediately after sample preparation in Lab 1 did not show a significant effect, while after 20 days the effects were significant (Table 2). This may indicate an effect of the time course. However, the differences in CuSO_4_ transmission values immediately after preparation and at age 20 days were in the same order of magnitude. Thus there is no strong evidence for an effect of the time course. Any such potential effects need further investigation.

## 5. Conclusion

The transmission of UV light for homeopathic preparations of CuSO_4_ was significantly lower than in controls. The transmission was also lower for both homeopathic preparations of SiO_2_ and S, but not significant.

UV transmission values between homeopathic preparations had a significantly higher variability compared to controls.

Thus, experimental evidence accumulates that highly diluted homeopathic preparations, that is, diluted beyond the Avogadro limit, exhibit particular physicochemical properties different from shaken pure solvent. The exact nature of these properties is not yet known; our current working hypothesis is an increase in the solvent's molecular dynamics for homeopathic preparations. All high-quality experimental data obtained so far by several independent working groups for different homeopathic preparations, involving studies with high- and low-field ^1^H NMR relaxation time, ^1^H-NMR-spectroscopy, and thermodynamics are compatible with this “dynamization hypothesis”.

## Funding

Software Foundation, Darmstadt, Germany; Wala Heilmittel GmbH, Boll, Germany; Dr Reckeweg & Co. AG, Bensheim, Germany. The sponsors had no influence whatsoever upon design, realization, evaluation and publication of the study.

## Figures and Tables

**Table 1:** The preparation and measurement process.

Substance	CuSO_4_ Series 1	CuSO_4_ Series 2	SiO_2_	S
Location of preparation	Lab 1	Lab 2	Lab 2	Lab 2
Clean room	No	Yes	Yes	Yes
Type of vessel	100 ml	500 ml	500 ml	500 ml
*N* controls/preparations	4/25	10/20	10/21	11/20
Spectroscopy instrument	*λ*14	*λ*3B	*λ*3B	*λ*3B
First measurements location	Lab 1	Lab 2	Lab 2	Lab 2
First measurements age (days)	0.1	20	7.5	6
Second measurements location	Lab 1			
Second measurements age (days)	61			

Two separate series of CuSO_4_ homeopathic preparations and controls were prepared, while there was one series of samples for sulfur (S) and quartz (SiO_2_). Lab 1 is a wet laboratory at the University of Illinois at Urbana Champaign and Lab 2 is a 100HEPA clean room at the National High Magnetic Field Laboratory, Tallahassee, FL. At Lab 1 a Perkin-Elmer *λ*14 and at Lab 2 a Perkin-Elmer *λ*3B UV-spectrometer were used. Samples of Lab 1 and Lab 2 were all measured by coupled plasma mass spectrometry at Lab 2.

**Table 2:** The results of the different measurements.

Substance	Location	Age (days)	Type	*N*	Wavelength range 190–290 nm				Wavelength range 215–290 nm			
					Mean ± SD	Difference ± SEM	*P t*-test	95% CI	Mean ± SD	Difference ± SEM	*P t*-test	95% CI
CuSO_4_	Lab 1	0.1	Control	4	86.5472 ± 0.0472	0.0528 ± 0.0297	0.1173	−0.0170 to 0.1226	89.4182 ± 0.0495	0.0451 ± 0.0290	0.1745	–0.0272 to 0.1175
		0.1	Preparation	25	86.4944 ± 0.0898				89.3730 ± 0.0756			
	Lab 1	61.0	Control	4	86.4850 ± 0.0459	0.0955 ± 0.0365	**0.0196**	0.0176 to 0.1734	89.4168 ± 0.0571	0.0656 ± 0.0356	0.1080	–0.0186 to 0.1498
		61.0	Preparation	25	86.3895 ± 0.1420				89.3512 ± 0.1060			
	Lab 2	20.0	Control	10	97.2199 ± 0.0503	0.0497 ± 0.0237	**0.0457**	0.0010 to 0.0983	98.9036 ± 0.0236	0.0351 ± 0.0116	**0.0053**	0.0113 to 0.0589
		20.0	Preparation	20	97.1703 ± 0.0783				98.8685 ± 0.0396			
SiO_2_	Lab 2	7.5	Control	10	97.4888 ± 0.1162	0.0645 ± 0.0498	0.2084	−0.0386 to 0.1675	99.0687 ± 0.0440	0.0323 ± 0.0187	0.0972	–0.0064 to 0.0710
		7.5	Preparation	21	97.4243 ± 0.1542				99.0364 ± 0.0572			
S	Lab 2	6.0	Control	11	97.2549 ± 0.3379	0.1257 ± 0.1355	0.3626	−0.1539 to 0.4054	99.0085 ± 0.0963	0.0281 ± 0.0388	0.4758	–0.0520 to 0.1082
		6.0	Preparation	20	97.1292 ± 0.3991				98.9804 ± 0.1152			
Pooled all	Labs 1 and 2	All	Control	39	0 ± 0.1903	0.0836 ± 0.0364	**0.0244**	0.0111 to 0.1562	0 ± 0.0604*	0.0456 ± 0.0129	**0.0006**	0.0199 to 0.0712
			Preparation	111	−0.0836 ± 0.2095				−0.0456 ± 0.0902*			
Pooled CuSO_4_	Labs 1 and 2	Various	Control	18	0 ± 0.0494*	0.0758 ± 0.0189	**0.0002**	0.0381 to 0.1136	0 ± 0.0395*	0.0544 ± 0.0142	**0.0003**	0.0260 to 0.0827
			Preparation	70	−0.0758 ± 0.1249*				−0.0544 ± 0.0897*			
Pooled Lab 2	Lab 2	Various	Control	31	0 ± 0.2129	0.0819 ± 0.0503	0.1076	0.0183 to 0.1821	0 ± 0.0626	0.0322 ± 0.0149	**0.0346**	0.0024 to 0.0620
			Preparation	61	−0.0819 ± 0.2547				−0.0322 ± 0.0769			

The column “Difference ± SEM” contains the difference between the homeopathic preparations and controls, which indicates the effect of homeopathic preparation on the UV-light transmission. The 95% CI does not include the zero value for statistically significant differences. There were significant differences between some of the CuSO_4_ homeopathic preparations and controls. Thus, the measurements at both locations that were done some days after preparation showed significant differences between homeopathic preparations and controls but not the measurements performed on the first day after preparation. The UV-transmission was also lower for both SiO_2_ and S, but not significant. The pooled data, once all data, once only the data for CuSO_4_ and once only for data from Lab 2 exhibit significant differences in transmission between homeopathic preparations and controls. Asterisk signifies a significant Levene's test, which indicates that the variance was significantly different between homeopathic preparation and control. Bold typeface indicates significance *P* < .05 .
